# A comparison of two hyperangulated video laryngoscope blades to direct laryngoscopy in a simulated infant airway: a bicentric, comparative, randomized manikin study

**DOI:** 10.1186/s12871-018-0580-y

**Published:** 2018-08-31

**Authors:** Marc Kriege, Nina Pirlich, Thomas Ott, Eva Wittenmeier, Frank Dette

**Affiliations:** Department of Anesthesiology, University Medical Center of the Johannes Gutenberg-University, Langenbeckstraße 1, 55131 Mainz, Germany

**Keywords:** Infant, Airway, Intratracheal intubation, Video laryngoscopy, Pediatric

## Abstract

**Background:**

In infants, securing the airway is time-critical because of anatomical and physiological differences related to airway management in children less than 1 year old. The aim of this study was to compare the time to ventilation using two different hyperangulated video laryngoscope blades with the time to ventilation via conventional direct laryngoscopy in a normal airway [NA] and in a simulated difficult airway [DA].

**Methods:**

This study was a comparative, bicentric, open-label, randomized controlled evaluation. An infant high-fidelity simulator (SimBaby™; Laerdal® Medical, Stavanger, Norway) was used, and two scenarios were proposed, as follows: NA and DA evoked with tongue edema and cervical collar. After theoretical and practical briefing, each participant compared in the two airway scenarios the novel King Vision™ Pediatric aBlade (KV) (Ambu® A/S, Bad Nauheim, Germany) video laryngoscope and the C-MAC™ D-blade Ped (DB) (Karl Storz® SE & Co. KG, Tuttlingen, Germany) video laryngoscope to conventional laryngoscopy using the Miller Blade (MiB) and the Macintosh Blade (MaB) in a random sequence.

**Results:**

Eighty physicians (65 AN and 15 PCCM staff) were included. In the NA scenario, the median [IQR] time to successful time to ventilation (TTV) was significantly shorter for the KV at 13 s [12–15 s] than for the MaB at 14.5 s [13–16 s], DB at 14.5 s [13–16] and MiB at 16 s [14–19] (*p* < 0.001). In DA, the KV also shortened TTV to 14 s [13–16], whereas TTV was 23 s with the MaB [20–26], 19 s with the DB [16–21], and 27 s with the MiB [22–31] (*p* < 0.001). There were no differences in first-pass intubation success rates (FPAs) between hyperangulated blades and direct laryngoscopes in NA. In DA, the hyperangulated blades enabled 92 (DB) to 100% (KV) FPAs compared with 65 (MiB) to 76% (MaB) for conventional laryngoscopy (*p* < 0.001).

**Conclusion:**

Video laryngoscopes with hyperangulated blades were associated with shorter TTV in normal and difficult infant airway situations. The higher FPAs of hyperangulated blades in DA may avoid desaturations and decrease adverse events in pediatric airway management.

**Electronic supplementary material:**

The online version of this article (10.1186/s12871-018-0580-y) contains supplementary material, which is available to authorized users.

## Background

Securing a pediatric airway is a critical skill for the anesthesiologist and pediatric intensive care physician [1]. Problems in airway management can lead to severe hypoxemia and are associated with prolonged mechanical ventilation in pediatric intensive care units (PICUs) [2]. Infants are significantly different from older children or adults with regard to their anatomical and physiological characteristics. The major anatomical differences in infants are large occiputs, larger tongues relative to their pharyngeal space, omega-shaped floppy epiglottises, and, most significantly, more cranially located larynges. Furthermore, the ranges of pathological processes typically seen in the pediatric population present unique anatomical or functional difficulties in airway management [3]. Consecutively, anesthesiologists and pediatric critical care medicine choose different airway tools to provide safe and effective control of the airway. Physiological differences include limited pulmonary reserve due to hemodynamic and respiratory compensation and urgent situations necessitating tracheal intubation place infants at increased physiological risk for adverse events. To optimize the time required for successful tracheal intubation during elective and urgent airway management, physicians can choose between direct or indirect laryngoscopy using the video laryngoscopy technique. Direct laryngoscopy requires alignment of the oropharyngeal-laryngeal axes, and these differences restrict optimal visualization of the glottis. The management of a difficult airway in infants with a weight of less than 10 kg has always been a challenge [4]. Fortunately, an unanticipated difficult airway is extremely rare in infants; however, the overall incidence of difficult laryngoscopy (Cormack & Lehane Class ≥ III) is significantly higher in patients < 12 months of age (4.7% vs. 0.7%) than in older children [1, 4].

Over the last 10 years, several studies have shown that video laryngoscopy is useful in pediatric anesthesia and critical care medicine [5, 6]. In contrast to conventional direct laryngoscopy, the video laryngoscopy technique enables visualization of the glottis without alignment of the oropharyngeal-laryngeal axes. The video laryngoscope blade offers field of vision of laryngeal structures similar to a Macintosh or Miller blade and provides 15- to 30-degree views to 60- to 90-degree views when used with a hyperangulated blade. Lifting the tongue and laryngeal structures using the direct laryngoscope may require a force up to approximately 1.5–3.0 kg [7]; in contrast, the force required when using a video laryngoscope with a hyperangulated blade design is only 0.4–1.3 kg, as measured in adult patients [7]. These advantages yield a smaller upward lifting force with less neck movement and alleviate stimulation of the oropharyngeal structures during laryngoscopy [7]. Selection of an adequate blade type may provide advantages of visualization of the glottis, higher first-pass intubation success rate (FPA) and optimization of the time to successful ventilation. The use of hyperangulated blades and conventional laryngoscope blades has not been extensively compared in pediatric patients to date, and such studies have been limited to a few small trials using these devices in normal pediatric airways in children aged approximately less than 1 year of age [6–8].

We designed a randomized controlled study involving a normal airway (NA) and simulated difficult airway (DA) with an infant high-fidelity simulator. We aimed to compare the performance of the novel King Vision™ Pediatric aBlade (KV) to that of the C-MAC™ D-Blade Ped (DB), both of which use hyperangulated video laryngoscopy blades, and two types of conventional direct laryngoscopes. The purpose of this study was to evaluate whether KV and DB can improve the time to successful ventilation over that achieved using conventional laryngoscopy in a simulated infant airway. As secondary endpoints, we collected the time to view, time to tracheal tube placement, FPA, glottis visualization and degree of ease or difficulty of tracheal intubation based on the Likert scale. We hypothesized that the use of hyperangulated blades would be superior to direct laryngoscopy in terms of time to ventilation in NA and DA.

## Methods

This study was a bicentric (Department of Anesthesiology and Center of Pediatric and Adolescent Medicine), comparative randomized study performed in the operating room (OR) and PICU at a tertiary university hospital. The ethics committee of the Medical Association of the state of Rhineland Palatine (Germany) approved this study (Registration No.: 837.384.14 (9623)). The order of devices used by the participants was determined by computerized randomization (http://www.random.org).

### Selection of participants

In all, 65 anesthesiologists and 15 practitioners of pediatric critical care medicine (PCCM) participated in this study. All were previously trained with video laryngoscopes and had sufficient clinical experience using the device (Table 1), with a lack of experience with the novel King Vision Pediatric aBlade. The expertise of the participating anesthesiologists and faculty of pediatric critical care medicine (PCCM) ranged from “beginner” (residents) to “expert” (consultants). Each physician was introduced to the study devices separately via a standardized instructional video by the principal investigator. After an introduction including handling as well the specifics of the device in NA, an intubation procedure was demonstrated. Subsequently, the participants had five attempts with each device to familiarize themselves and obtain an adequate learning curve (8) with each device before the evaluation started. As there were four devices and two airway scenarios, each participant performed a total of 8 intubations.

**Table 1:** Demographics and level of experience

Provider analysis	Anesthesiologists (*n* = 65)	PCCM staff (*n* = 15)	*p*-value
Status			
Residents/Fellows/Consultants	51/11/3	6/4/5	0.33
Experience			
Practice experience (months)	30 (6–180 [21–48])	36 (6–180 [24–96])	0.43
Airway management in children (< 6 y)			
< 10 applications	6 (9%)	0	0.22
< 50 applications	21 (32%)	4 (26%)	0.67
< 100 applications	26 (40%)	2 (13%)	0.05
> 100 applications	12 (19%)	9 (61%)	0.001
Airway management in infants/children			
< 5 kg	37 (57%)	15 (100%)	0.001
< 10 kg	62 (95%)	15 (100%)	0.39
< 20 kg	65 (100%)	15 (100%)	1.0
Experience in VL (applications)			
< 10	0	0	
< 25	4 (6%)	5 (33%)	0.002
< 50	13 (22%)	1 (7%)	0.22
> 100	48 (74%)	9 (60%)	0.28
Experience in DL (applications)			
< 50	0	0	
< 100	2 (3%)	4 (27%)	0.001
< 500	15 (23%)	5 (33%)	0.4
< 1000	31 (48%)	4 (27%)	0.14
> 1000	17 (26%)	2 (13%)	0.29

Data are presented as medians (range [IQR] and absolute numbers (proportion)

### Setting and interventions

**Fig. 1:**
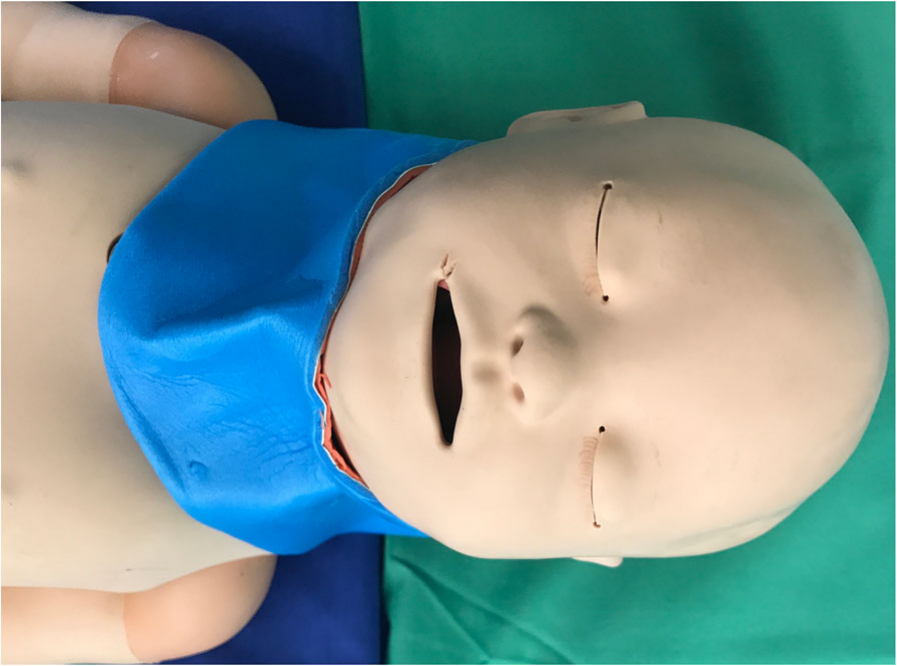
Standardized position of the SimBaby with a shoulder roll to elevate the shoulders and a soft donut to stabilize the head

The study was conducted in an antechamber of the OR PICU with the high-fidelity simulator positioned at the head of a stretcher (Fig. 1). Participants were allowed to adjust the stretcher or chair to a comfortable height. A pediatric high-fidelity Simulator SimBaby™ (Laerdal® Medical, Stavanger, Norway) was built to resemble a 65-cm long healthy baby and was capable of creating easy and difficult airway situations. The SimBaby is a realistic airway that includes crying and gurgling sounds, cyanosis, tongue edema, pharyngeal obstruction, chest excursion, and breath sounds. In relation to a real infant derived from growth charts of the WHO, this length corresponds to that of a 6- to 8-month-old infant when looking at the area between the 5th and 95th growth percentile [9, 10]. We evaluated the devices under two airway scenarios as follows: (i) normal (NA) and (ii) difficult intubation condition (DA) created through tongue edema and a cervical collar (Fig. 1; SAM® Splint™, SAM Medical Products, Wilsonville, OR, USA) to reduce the mouth opening from 2.6 mm before to 1.5 mm after adjustment of an extrication collar to inhibit neck movement.

**Fig. 2:**
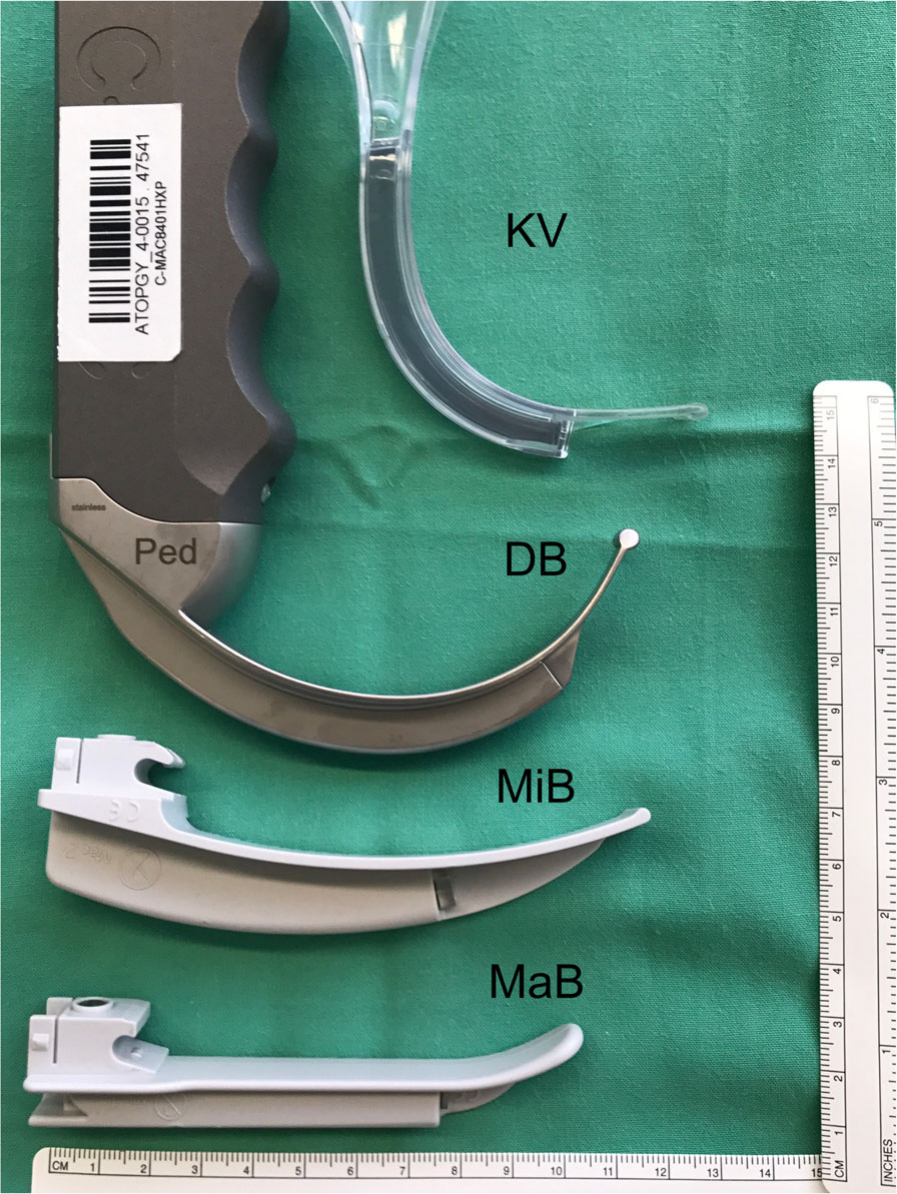
In order from top to bottom: KV #2 standard blade, DB, Macintosh blade #2 and Miller blade #1

We chose to compare two video laryngoscopes that use a hyperangulated blade with two conventional direct laryngoscopes. The King Vision™ Pediatric aBlade (KV) (Ambu® GmbH, Bad Nauheim, Germany) is a novel video laryngoscope used in a clinical setting in pediatric patients and has not been evaluated under various airway conditions. The KV has a built-in video screen and is available in three pediatric sizes, #1–3 (≥ #2 has a channeled and standard disposable blade with a 37.8-degree angulated blade). In this study, we used a KV #2 standard blade (Fig. 2). The C-MAC™ D-Blade Ped (DB) (Karl Storz®, Tuttlingen, Germany) is a compact system consisting of a monitor, electronic module and interchangeable video laryngoscope blade. The DB has an increased curvature with a 75-degree blade angulation. The manufacturer recommends its use in infants or small children weighing 9.8 to 22 kg. The DB was specifically developed to manage anatomically difficult airway conditions such as those encountered in Down syndrome or Pierre-Robin syndrome. The conventional direct laryngoscopes included the Miller straight blade #1 (MiB) and a Macintosh curved blade #2 (MaB) with a standard laryngoscope handle for pediatric patients. All sizes of the blades were tested prior to the study; they are shown in Fig. 2.

Per the recommendation of the manufacturer of the SimBaby, all intubations were performed with a 3.5-mm internal diameter cuffed endotracheal tube (ET). During intubation using one of the video laryngoscopes, a malleable stylet was inserted into the ET, which was angulated into a hockey-stick shape (distal end of ET angulated 90°). When using a conventional direct laryngoscope, the rigidity of the ET was provided by a malleable stylet (Mallinckrodt® satin slip, 2.6 mm). The SimBaby was placed in a standardized position, and a shoulder roll and a soft donut-shaped foam headrest were used to support the head in all cases (Fig. 1).

### Methods of measurements

Demographic analysis was collected for all participants concerning the clinical level of airway management experience with children ≤5 years of age, the time since their most recent intubation in each age group (newborns, infants, and small children) and experience with video laryngoscopy (VL) and conventional direct laryngoscopy in real patients [11]. Experienced users (consultants) were defined as those with performance experience in pediatric tracheal intubation exceeding 100 infants. Inexperienced users were defined as those with experience in less than 100 tracheal intubations in infants [6].

The time interval between the blades passing the gums to confirmation of the best glottic view was recorded as the time to best view (TTBV). The participant announced the Cormack & Lehane Classification (C&L) and the percentage of glottic opening (POGO) score once the best glottic exposure was obtained [12, 13]. External laryngeal manipulations (ELMs) [14], such as the BURP (backwards, upwards and rightwards pressure) maneuver, can be applied to improve the view of the glottis to achieve a C&L I or II. The time to place (TTP) the ET was defined as the time when the first black mark on the ET was threaded between the vocal cords. The time to ventilation (TTV) in seconds (s) was defined as the time from when the blade tip passed the gums of the SimBaby to the point of confirmation of the first visible chest rise of the simulator. Time was measured using a stopwatch. Study personnel were positioned on the right site of the SimBaby.

Failed intubation was defined as follows: a) an elapsed intubation time of more than 40 s in accordance with the current recommendations and in the time period of previously published data [6, 15, 16]; b) failed tracheal placement of the ET (i.e., esophageal); and c) removal of the device/repositioning from the oral cavity without advancing the ET. If the first attempt failed, the provider made a second laryngoscopy attempt with the same device. A total of two laryngoscopy attempts were allowed. If DL failed, the clinician changed to a prescribed rescue technique with KV. If VL failed after two attempts, the clinician was advised to proceed with DL. The limitation of two intubation attempts and choice of an alternative technique has been recommended in several studies and was chosen in accordance with clinical standards [17]. After each intubation, the physician was asked to rate the degree of difficulty of intubation using a 5-point Likert scale (1 = very easy to 5 = very difficult) for each blade [18].

### Outcome measures

Our primary study objective was to determine whether there was a difference in TTV, demonstrated by bilateral lung inflation of the manikin with positive pressure via bag-valve ventilation, between the KV and the DB, MiB and MaB. Secondary outcomes were the TTBV and the TTP, visualization of laryngeal structures using the C&L Classification and the POGO score, FPA, the use of ELMs and the degree of difficulty.

### Statistical analysis

A statistical analysis of the data was subsequently undertaken. The Shapiro-Wilk test was used to test the assumption of normal distribution (*p* > 0.1). Normally distributed data are presented as the means (SD) and were analyzed using an independent t-test for unequal variances. Non-normally distributed interval and ordinal data are reported as medians (interquartile range [IQR]); results were compared among groups using the Wilcoxon-Mann-Whitney test. Categorical variables are presented as counts; results were evaluated using the chi-square test. TTV data were compared between groups using the log-rank test. All recorded data were documented using a controlled case report form. Devices were randomized to treatment groups using the GraphPad QuickCalcs Web site: http://www.graphpad.com/quickcalcs/randmenu (accessed January 2015). GraphPad Prism (Vers. 6.0 for MAC; GraphPad Software, San Diego, CA, USA) was used for all statistical analyses. Differences in TTV were considered statistically significant if the *p*-value was less than 0.05.

## Results

### Characteristics of study subjects

From July to August 2017, a total of 80 physicians (anesthesiologists, *n* = 65; PCCM staff, *n* = 15) eligible to participate in this study were included (Table 1). These 80 participants performed 640 intubation attempts. The faculty of PCCM had more experience in airway management in children less than 6 years of age (*p* < 0.001) and infants with a weight < 5 kg (*p <* 0.001). Otherwise, the anesthesiologists had more overall experience in VL (> 25 applications prior to the study; *p* = 0.002) and DL (> 100 applications prior the study; *p* < 0.001) compared to PCCM. Demographics and the level of experience of all subjects are presented in Table 1.

### Main results

**Fig. 3:**
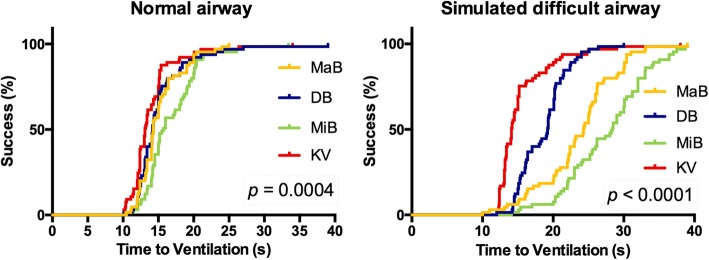
Kaplan-Meier plots of the time to ventilation for all four devices in (left) the normal airway situation and (right) a difficult airway

For the primary outcome, the median duration for TTV was shorter for the KV at 13 s [12–15] than for the MaB at 14.5 s [13–16], the DB at 14.5 s [13–16], and the MiB at 16 s [14–19]; *p* < 0.001) in the NA scenario. In DA, the KV similarly significantly shortened TTV to 14 s [13–16], whereas TTV was 23 s using the MaB [20–26], 19 s using the DB [16–21], and 27 s using the MiB [22–31]; *p* < 0.001). Both groups (anesthesiologists and PCCM staff) were similar with respect to the time required to obtain TTV with all four devices (*p* = 0.5). Furthermore, TTV was shorter when using the hyperangulated blades for both experienced and inexperienced physicians in the NA/DA scenario (*p* < 0.001). Study outcomes for the various blades are outlined in Table 2 for the NA scenario and in Table 3 for the DA scenario. Figure 3 present the Kaplan-Meier plots of the TTV with all four devices in the NA scenario and DA scenario, respectively.

**Table 2:** Study results by laryngoscope blades in the NA scenario

*p*-values of Pairwise Differences									
Outcomes	MaB	DB	MiB	KV	DB vs. MaB	DB vs. MiB	KV vs. MaB	KV vs. MiB	KV vs. DB
Time ^a^ Sequences (s)									
TTBV	5.5 [5–7]	5 [4–6.5]	7 [5–8]	5 [3.5–6]	0.01	< 0.001	< 0.001	< 0.001	0.02
TTP	10.25 [9–11.5]	10 [9–11.5]	12 [10–14.5]	9.5 [8.5–11]	0.85	< 0.001	0.02	< 0.001	0.002
TTV	14.5 [13–16]	14.5 [13–16]	16 [14–19]	13 [12–15]	0.75	0.001	0.002	< 0.001	< 0.001
No. attempts^b^									
1st	80/80 (100%)	79/80 (98%)	78/80 (97%)	80/80 (100%)	0.31	0.56	> 0.99	0.15	0.31
2nd	0	1/80 (2%)	2/80 (3%)	0		0.56			
Failed	0	0	0	0					
Glottic view									
C&L 1/2/3^b^	67/13/0	78/2/0	57/23/0	79/1/0	0.007	< 0.001	0.001	< 0.001	> 0.99
POGO (%)^a^	90 [90–100]	100 [100]	95 [80–100]	100 [100]	< 0.001	< 0.001	< 0.001	< 0.001	0.84
ELM^b^	5/80 (6%)	0/80 (0%)	11/80 (14%)	1/80 (1%)	0.02	< 0.001	0.09	0.002	0.32
BURP	0	0	6/11 (54%)	1/1 (100%)					
Neck extension	5/5 (100%)	0	7/11 (63%)	0					
Degree of ^a^ difficulty (1–5)	1 [1–2]	1 [1–1]	1 [1–2]	1 [1–2]	0.44	< 0.001	< 0.001	0.9	< 0.001

^a^Medians and [IQR] are shown. Pairwise differences among devices were evaluated using the signed-rank test

^b^Absolute numbers (proportion) are shown. Pairwise differences among devices were evaluated using the chi-square test

**Table 3:** Study results by laryngoscope blades in the DA scenario

*p*-values of Pairwise Differences									
Outcomes	MaB	DB	MiB	KV	DB vs. MaB	DB vs. MiB	KV vs. MaB	KV vs. MiB	KV vs. DB
Time ^a^ Sequences (s)									
TTBV	11 [8.5–14]	8 [6.5–9]	13.5 [9–15.5]	6.5 [5–7]	< 0.001	< 0.001	< 0.001	< 0.001	< 0.001
TTP	17.5 [15–20]	14 [11–15]	20.5 [16.5–24]	10 [9.5–12]	< 0.001	< 0.001	< 0.001	< 0.001	< 0.001
TTV	23 20–26]	19 [16–21]	27 [22–31]	14 [13–16]	< 0.001	< 0.001	< 0.001	< 0.001	< 0.001
No. attempts^b^									
1st	61/80 (76%)	74/80 (92%)	52/80 (65%)	80/80 (100%)	0.004	< 0.001	< 0.001	< 0.001	0.01
2nd	16/80 (20%)	6/80 (8%)	23/80 (29%)	0	0.29	0.26			
Failed	3/80 (4%)	0	5/80 (6%)	0					
Glottic view									
C&L 1/2/3^b^	19/60/1	61/19/0	7/70/3	77/3/0	< 0.001	< 0.001	< 0.001	< 0.001	< 0.001
POGO (%)^a^	40 [30–60]	90 [90–100]	40 [30–60]	100 [90–100]	< 0.001	< 0.001	< 0.001	< 0.001	< 0.001
ELM^b^	3/80 (4%)	1/80 (1%)	4/80 (5%)	0/80 (0%)	0.31	0.17	0.08	0.04	0.32
BURP	0	0	0	0					
Neck extension	3/3 (100%)	1/1 (100%)	4/4 (100%)						
Degree of^a^ difficulty (1–5)	2 [2–3]	1 [1–2]	3 [3–3]	1 [1–1]	< 0.001	< 0.001	< 0.001	< 0.001	0.001

^a^Medians and [IQR] are shown. Pairwise differences among devices were evaluated using the signed-rank test

^b^Absolute numbers (proportion) are shown. Pairwise differences among devices were evaluated using the chi-square test

The overall intubation success rates were higher for the KV 80/80 (100%) in both airway scenarios performed by anesthesiologists and PCCM staff (*p* < 0.001). The number of attempts with each device is listed in Table 2 for the NA scenario and in Table 3 for the DA scenario. A proportion of 8/320 (2.5%) participants had > 2 failed attempts with the DL in the DA scenario (*p* < 0.001). All were successfully intubated with the KV. The reason for a second attempt in the NA scenario was a timeout > 40 s in 3/320 (0.9%) cases, and the reasons for a second attempt in the DA scenario were a timeout in 28/320 (9%) of the trials and removal of the blade in 17/320 (5.3%) of the trials (*p* = 0.1).

In the NA scenario, we obtained a better view of the glottis with hyperangulated blades than with the conventional laryngoscopes (*p* < 0.001). In the DA scenario, the KV enabled a better glottic view (C&L I) 80/80 (100%) than was achieved using the DB 74/80 (92%), MaB 61/80 (76%) and MiB 52/80 (65%; *p* < 0.001).

After completing the series of simulated intubations, the participants rated the degree of difficulty in the NA scenario as lower for the DB than for the MaB, MiB and KV (*p* < 0.001). In contrast, in the DA scenario, the KV was rated easier to use compared with the DB, MaB or MiB (*p* = 0.001).

## Discussion

In the present study, a shorter TTV was achieved using video laryngoscopes with a hyperangulated blade in a group of 640 intubation attempts in an infant DA. In particular, TTV was shorter in both airway situations when applying the novel KV. The FPA, visualization of the glottis and subjective assessment in this comparative study were superior to those of conventional direct laryngoscopy. To date, only one study compared the novel KV with the traditional MiB in elective surgeries performed in children < 2 y of age [19]. This study is one of the first studies comparing the KV with another hyperangulated blade and direct laryngoscopy in a simulated infant airway.

The demographics of the participants were comparable to those in other studies investigating the MaB, DB or MiB [6, 10, 12, 20–22] in pediatric airways. In contrast to other studies comparing experienced and inexperienced practitioners [6, 9–11, 19, 21, 22], this study demonstrated that TTV was similar between the groups. However, in this study, there were no institutional differences in FPA or TTV among the blades or airway scenarios. This likely reflects a familiarity with the direct and video laryngoscopy techniques. Furthermore, every participant only had 5 attempts with the novel KV or DB prior to the study.

A number of new devices have been introduced to facilitate tracheal intubation in pediatric patients with normal and difficult airways, with reports describing varying success rates and experience in pediatric airway management [10, 11, 22]. In these experiments, manikins were used in various simulation scenarios, including tracheal intubation. In addition to their use in training, they have also been used in various clinical research projects to demonstrate the efficacy of one device over another. SimBaby™ has been used to teach clinical and decision-making skills while mimicking patient care scenarios [9, 10, 23]. Tracheal intubation using a video laryngoscope might be easier to learn than conventional direct laryngoscopy [8, 9]. The success rates vary considerably, and the proportion of patients in whom the glottic opening can be visualized but the ET cannot be inserted into the trachea is high [20, 21, 24]. Several study results have noted that intubation time is longer when using a video laryngoscope with a hyperangulated blade [6, 9, 21]. Surprisingly, TTV was significantly shorter for the novel KV blade in the NA (1.5 s) and DA (13 s) scenarios than for the other devices, which influence the occurrence of systemic complications (e.g., hypoxemia). TTV was longer for the MaB and MiB than for hyperangulated blades relative to the results of studies looking at two conventional blades; however, the values were within the range of published data [6, 9, 23]. In two studies that evaluated children aged < 2 years of age, TTV was shorter for the MiB [19, 22] than for the KV. Straight blades are often recommended for use in neonates and infants to lift the relatively large and floppy epiglottis [22]. These blades avoid a situation in which the hyoid bone cannot be displaced forward and the epiglottis continues to obstruct a view of the larynx. In summary, a good laryngeal view with the intubating device did not equate with ease of intubation. Otherwise, a curved MaB provides more room in the oropharynx to maneuver the ET. The time to visualization of the glottis and successful ventilation were also comparable to the results reported in another study [19]. However, the measurements differed between these studies in which the practitioner took hold of the handle of the device until the cuff of the ET was inflated [9, 20, 24] or until confirmation of the first inflection of the end-tidal capnogram on the anesthesia respirator [6, 19]. In three studies, time measurement stopped when successful lung expansion occurred [9, 20, 21]. All intubations were successfully performed using the KV on FPAs. Comparing TTV or FPA using the DB is difficult because the literature is quite sparse with regard to the use of the DB in pediatric patients or in a suitable manikin. The prolonged intubation time and lower success rate in DB and conventional direct laryngoscopes might be explained as follows: first, the anterior view angle of 55 degrees achieved using the KV differs from the half-moon shaped DB (embedded optical lens with aperture angle of 80°); furthermore, the length differs between the two hyperangulated blades (KV, 7 cm vs. DB, 10 cm). Additionally, it is generally thought that the position of the head has an influence on the laryngeal view [6]. We used a standardized position for the SimBaby in which a shoulder roll elevated the shoulders and a small donut was used to support the head. The goal was to align the oral, pharyngeal, and tracheal axes to facilitate tracheal intubation.

As a secondary outcome, visualization of the glottis was significantly easier with VL in both scenarios. These data are consistent with previously published data in simulated pediatric patients in whom the laryngeal view was found to be better with VL [6, 9, 10, 19]. In contrast to other studies comparing hyperangulated blades with DL, we found a higher FPA and overall success rate when using the KV or DB [11, 15, 21]. In fact, a higher FPA was shown with the KV in DA compared to the first study that evaluated the KV in a normal pediatric airway [19]. These data are comparable to that in study with manikins where the results vary with the operator’s prior experience and familiarity with the equipment, institutional preferences, and how well the manikin simulates a real patient [11, 20]. Finally, when considering the number of ELMs, which were slightly higher with direct laryngoscopy, and the participants’ subjective difficulty scores, which were also higher for these devices, our data agree with those reported on the management of adult patients [18]. This fact may be derived from the absence of prior experience of the subjects with the DB and KV or with the specific characteristics of the conventional blades and the infant manikin’s airway anatomy.

### Study limitations

Our study has several limitations. First, we used two different conventional and two different hyperangulated video laryngoscopy blade sizes in an infant simulator. We cannot assure that the simulated clinical conditions truly reproduce the real patient’s conditions. Second, this comparative study might have been limited by the heterogeneous experiences of the providers (e.g., experiences in anesthesia practice or pediatric critical care medicine and in video laryngoscopy techniques). We feel, however, that this approach is more clinically relevant than are the study results, reflecting the experience of a small team of anesthesiologists who are highly skilled in airway management. Because of the design of this study, the protocol did not allow blinding of the operators using the different blades. We compared the KV and DB video laryngoscopes (hyperangulated blades) to two direct laryngoscopes with the straight blade (MiB) or a curved blade (MaB) that are used by practitioners with various levels of experience with the four devices. We chose to compare a curved to a straight standard laryngoscope for the following reasons: The most popular curved Macintosh blade differs in angulation from the KV/DB blade, and comparing the two represents a comparison of three disparate blades. Traditional clinical teaching suggests that the straight blade offers the best glottis exposure in infants because of the higher location of the glottis. The lower peak lifting force on the base of the tongue using the hyperangulated video laryngoscopy blades might be associated with a higher success rate in the SimBaby. Finally, TTV might differ in real infants because the retroglossal airspace volume of the SimBaby is 5.3 ± 0.4 cm^3^ instead of the 1.9 ± 0.8 cm^3^ observed in infants [25].

## Conclusion

Use of a hyperangulated video laryngoscope blade, especially the novel King Vision Pediatric aBlade, resulted in significantly faster TTVs and higher FPAs than were achieved using conventional direct laryngoscope blades. VL in general may offer promise for intubation of NA and DA in children. The use of a hyperangulated blade optimizes the placement of ETs by anesthesiologists and PCCM staff in simulated infant airways. Further studies aimed at comparing various video-based laryngoscope blade designs used in infants or children with different types of airway problems will be useful for assisting anesthesiologists and pediatricians in selecting the most appropriate device to use in each individual clinical scenario.

## Additional file

Additional file 1: Availibility data. (XLSX 99 kb)
